# Characterization of *Bacillus cereus* Group Isolates From Human Bacteremia by Whole-Genome Sequencing

**DOI:** 10.3389/fmicb.2020.599524

**Published:** 2021-01-12

**Authors:** Angelica Bianco, Loredana Capozzi, Maria Rosa Monno, Laura Del Sambro, Viviana Manzulli, Graziano Pesole, Daniela Loconsole, Antonio Parisi

**Affiliations:** ^1^Istituto Zooprofilattico Sperimentale della Puglia e della Basilicata, Foggia, Italy; ^2^Department of Basic Medical Sciences, Neurosciences and Sense Organs, University of Bari “Aldo Moro”, Bari, Italy; ^3^Dipartimento di Bioscienze, Biotecnologie e Biofarmaceutica, University of Bari “A. Moro”, Institute of Biomembranes, Bioenergetics and Molecular Biotechnologies of the National Research Council and Consorzio Interuniversitario Biotecnologie, Bari, Italy; ^4^Department of Biomedical Sciences and Human Oncology, Hygiene Unit, University of Bari “Aldo Moro”, Bari, Italy

**Keywords:** *Bacillus cereus* group, whole-genome sequencing (WGS), ANIBlast, BTyper, virulence factors

## Abstract

Members of the *Bacillus cereus* group are spore-forming organisms commonly associated with food poisoning and intestinal infections. Moreover, some strains of the group (i.e., *B. cereus* sensu stricto and *Bacillus thuringiensis*) can cause bacteremia in humans, mainly in immunocompromised individuals. Here we performed the genetic characterization of 17 human clinical strains belonging to *B. cereus* group isolated from blood culture. The whole-genome sequencing (WGS) revealed that the isolates were closely related to *B. cereus* sensu stricto and *B. thuringiensis*–type strain. Multilocus sequence typing analysis performed on the draft genome revealed the genetic diversity of our isolates, which were assigned to different sequence types. Based on *panC* nucleotide sequence, the isolates were grouped in the phylogenetic groups III and IV. The *NHE*, *cer*, and *inhA* gene cluster, *entA*, *entFM*, *plcA*, and *plcB*, were the most commonly detected virulence genes. Although we did not assess the ability to generate biofilm by phenotypic tests, we verified the prevalence of biofilm associated genes using an *in silico* approach. A high prevalence of *pur* gene cluster, *xerC*, *clpY*, *codY*, *tasA*, *sipW*, *sinI*, and *sigB* genes, was found. Genes related to the resistance to penicillin, trimethoprim, and ceftriaxone were identified in most of the isolates. Intriguingly, the majority of these virulence and AMR genes appeared to be evenly distributed among *B. cereus* s.s. isolates, as well as closely related to *B. thuringiensis* isolates. We showed the WGS represents a good approach to rapidly characterize *B. cereus* group strains, being able to give useful information about genetic epidemiology, the presence of virulence and antimicrobial genes, and finally about the potential hazard related to this underestimated risk.

## Introduction

*Bacillus cereus* sensu lato (*B. cereus* s.l.), known also as *B. cereu*s group, consists of at least 12 spore-forming Gram-positive bacteria that are optionally motile and facultative anaerobic saprophyte (Liu et al., 2017). *B. cereus* group is widespread in nature as spores and vegetative cells. The spores are resistant to extreme environmental conditions (i.e., heat, freezing, drying, radiations) and germinate when they come into contact with organic matter or within an animal host (Bottone, 2010). The group includes *B. cereus* sensu stricto (s.s.), which is responsible for both diarrheal and emetic human gastrointestinal syndromes and extraintestinal infections; *Bacillus thuringiensis*, an entomopathogen characterized by the production of crystal inclusions (containing insecticidal proteins); *Bacillus anthracis*, the agent of anthrax in humans and animals; *Bacillus mycoides* and *Bacillus pseudomycoides*, both of which are characterized by rhizoidal colonies on solid media and have not been described as food poisoning agents; *Bacillus weihenstephanensis*, a psychrotolerant bacterium; *Bacillus toyonensis*, which exhibits both probiotic and hemolytic properties; psychrotolerant and cytotoxic *Bacillus wiedmannii* (Miller et al., 2016); thermotolerant *Bacillus cytotoxicus*, which is responsible for occasional infections (Guinebretière et al., 2013); finally, the recently identified *Bacillus paranthracis*, *Bacillus pacificus*, *Bacillus tropicus*, *Bacillus albus*, *Bacillus mobilis*, *Bacillus Luti*, *Bacillus proteolyticus*, *Bacillus nitratireducens*, *Bacillus paramycoides*, *Bacillus gaemokensis*, *Bacillus manliponensis*, *Bacillus bingmayongensis*, and *Bacillus fungorum* (Liu et al., 2015, 2017, 2020). *B. cereus* s.l. is mainly responsible for two types of intoxication: the emetic gastrointestinal syndrome characterized by vomiting, strongly associated with rice and derived products (Johnson et al., 1983), and the diarrheal syndrome, characterized by aqueous diarrhea associated with abdominal pain. *B. cereus* s.l. is also involved in several non-gastrointestinal-tract clinical infections. The spectrum of syndromes includes fulminant septicemia, central nervous system involvement (meningitis and brain abscesses), gas gangrene–like infections (Bottone, 2010), progressive pneumonia (Hoffmaster et al., 2006), severe ocular infections such as endophthalmitis, and bacteremia in preterm neonates (Hilliard et al., 2003). Most commonly infected people are immunosuppressed patients (Goldstein and Abrutyn, 1985; Bryce et al., 1993), patients undergoing surgery, intravenous drug users, and patients with indwelling catheters (Hernaiz et al., 2003). The *B. cereus* catheter-related infections are generally caused by the formation of biofilm on biomedical devices (Ash et al., 1991; Kuroki et al., 2009; Liu et al., 2015). Hospital environment sources of *B. cereus* group include air filtration and ventilation equipment (Bryce et al., 1993), fiber-optic bronchoscopy equipment (Goldstein and Abrutyn, 1985), intravenous catheters (Hernaiz et al., 2003), and alcohol-based hand wash solutions (Hsueh et al., 1999). In recent years, it has been speculated that the gastrointestinal tract can act as a potential source of *B. cereus* strains acquired from an exogenous source (food, water, environment), which can invade the gastrointestinal tract, cause mucosal necrosis, and spread to other organs through the bloodstream (Bottone, 2010). Despite the low number of reports, also *B. thuringiensis* has been reported to be involved in gastrointestinal diseases (Jackson et al., 1995). Some *B. thuringiensis* strains are able to produce enterotoxins (Damgaard et al., 1997; Ghelardi et al., 2007) and possess genes known to be involved in the pathogenesis of *B. cereus* infections (Kreig and Lysenko, 1979; Hsieh et al., 1999).

The discrimination between pathogenic and non-pathogenic *B. cereus* group isolates has become a matter of public health. However, the close genetic relationship existing among the members of *B. cereus* group makes their identification to species level difficult, indicating that they have diverged from a common evolutionary lineage (Orrett, 2000; Liu et al., 2015).

Phenotypic and biochemical methods, as well as molecular methods, such as 16S rDNA or 23S rDNA sequencing, may not have sufficient discriminatory power to differentiate between members of the group (Kato et al., 2014; Yan et al., 2017). For these reasons, some other genetic loci have been selected as markers to differentiate between pathogenic and harmless *B cereus* group strains. Among these is the *rpo*B housekeeping gene (Caamaño-Antelo et al., 2015) or the pantoate-beta-alanine ligase gene (*pan*C) (Schmid et al., 2016; Warda et al., 2016), which classifies *B. cereus* isolates in seven phylogenetic groups (I to VII) (Guinebretière et al., 2008). Moreover, different schemes have been standardized for multilocus sequence typing (MLST), defined as TH (Tourasse et al., 2006), P (Priest et al., 2004), K, H (Helgason et al., 2004), and CS (Candelon et al., 2004; Sorokin et al., 2006)^1^. Recently, it has been shown that these methods are largely congruent in the *B. cereus* s.l. genomospecies attribution (Carroll et al., 2020). In order to evaluate the presence of the main virulence factors, generally polymerase chain reaction (PCR) amplifications are performed for the identification of seven enterotoxigenic among the *B. cereus* virulence genes: hemolysin BL (*hbl*A, *hbl*C, *hbl*D), enterotoxin non-hemolytic (*nhe*A, *nhe*B, *nhe*C); cytotoxin K (*cyt*K); enterotoxin FM (*ent*FM), enterotoxin S (*ent*S), and emetic toxin (*ces*) (Fricker et al., 2007; Owusu-Kwarteng et al., 2017).

The aim of this study was to characterize *B. cereus* s.l. isolated from 17 samples of blood cultures from hospitalized patients using different approaches. The presence of the genes associated with virulence and antimicrobial resistance (AMR) was checked by whole-genome sequencing (WGS) in all the isolates. Further, the *in vitro* sensitivity to antimicrobials of *B. cereus* isolates has also been evaluated.

## Materials and Methods

Seventeen Gram-positive *Bacillus* spp. isolated from blood culture collected from 17 epidemiologically non-related patients in the period 2004–2018 in a teaching hospital of Bari, Southern Italy, were studied. Blood culture analysis was performed by BacT/Alert system with FAN Plus Aerobic medium (bioMérieux, Marcy l’Etoile, France). When a positive bottle was flagged, a Gram stain of the broth was performed, and a portion of the fluid was subcultured on PolyViteX agar and on Columbia agar with 5% sheep blood (bioMérieux, Marcy l’Etoile, France). Identification of isolates was performed by VITEK 2 Automated system (bioMérieux, Marcy l’Etoile, France). For each patient, the strain belonging to *Bacillus* spp. was isolated from three separate blood cultures.

### Matrix-Assisted Laser Desorption Ionization–Time of Flight Spectrometry

Prior to matrix-assisted laser desorption/ionization–time of flight (MALDI-TOF) analysis, the isolates were cultivated on Columbia blood agar for 18–24 h at 37°C. After incubation, a sterile wooden tip was used to pick an isolated bacterial colony freshly grown and then smearing a thin film onto a 96-polished steel target plate (Bruker Daltonik GmbH, Germany) (direct transfer sample preparation procedure). Microbial films were overlaid with 1 μL α-cyano-4-hydroxycynnamic acid–matrix solution (Bruker Daltonik GmbH, Germany), prepared following the instruction for use and with final concentration of 10 mg/mL.

The sample-matrix mixture was dried at room temperature and subsequently inserted into the system for data acquisition. The mass spectra were generated using a MALDI-TOF system Microflex LT/SH^TM^ (Bruker Daltonik GmbH, Germany), which was operated in linear positive mode covering the molecular weight range of 2,000–20,000 Da. Each strain was applied to 10 spots, and each spot was hit 240 shots each in several points with a pulsed nitrogen laser beam operating at 337 nm, with a frequency equal to 60 Hz. Acceleration voltage was set at 20 kV, and the instrument was calibrated in the range of 3,637.8 and 16,953.3 Da using *Escherichia coli* DH5α (BTS, Bruker Daltonik GmbH, Germany). The data were processed automatically by the instrument software MBT Compass 4.1.70.1 database version 7.0.0.0 (Bruker Daltonik GmbH, Germany), and the spectra were compared with reference libraries for bacterial identification matching. The degree of correspondence between the test spectrum and the reference spectra in the database determines the attribution of the logarithmic score value (0–3.0). When a logarithmic score was < 1.7, the spectrum was reported as “not reliable identification,” indicating that it could not identify the genus or species of the strain. A logarithmic score between 1.7 and 2.0 indicates that identification could be reliable only at the genus level, whereas a logarithmic score between 2.0 and 3.0 indicates that identification could be reliable at the species level of the organism.

### Antibiotics Susceptibility Testing

The minimum inhibitory concentration (MIC) was used to determine antimicrobial susceptibility *in vitro* according to the Clinical and Laboratory Standards Institute (CLSI), as previously reported (Manzulli et al., 2019). The antibiotics tested were gentamicin, ceftriaxone, penicillin G, clindamycin, chloramphenicol, vancomycin, linezolid, cefotaxime, tetracycline, erythromycin, rifampin, amoxicillin, ciprofloxacin, doxycycline, and trimethoprim.

The CLSI breakpoints (μg/mL) for penicillin, ciprofloxacin, doxycicline, tetracycline, and cephems were those suggested for *B. cereus*, whereas for the other antimicrobials, interpretative criteria for *Staphylococcus* spp. were used according to CLSI guidelines M45-A2 (2011) and M100 (2017) (Sarker et al., 2007; Weinstein, 2018). *Staphylococcus aureus* ATCC 29213 and *E. coli* ATCC 25922 were used as control strains.

### Whole-Genome Sequencing and Typing

Genomic DNA was extracted from the *B. cereus* s.l. isolates using DNeasy Blood and Tissue Kit (Qiagen, Hilden, Germany), according to the manufacturer’s protocol. DNA quality and concentrations were estimated by Qubit Fluorometer using Qubit dsDNA HS Assay (Thermo Fisher Scientific). For each isolate, paired-end genomic libraries were prepared using Nextera DNA Flex Library preparation kit (Illumina, San Diego, CA, United States). Sequencing was performed using MiSeq Reagent Kit v2 (2 × 250 bp) on Illumina MiSeq platform (Illumina, San Diego, CA, United States). The paired-end raw reads were trimmed using Trimmomatic (Galaxy Version 0.36.6) (Bolger et al., 2014), and then the draft genomes were assembled by SPAdes 3.12.0 (Bankevich et al., 2012). Assembled genomes were submitted to BTyper tool (version 2.3.2) (Carroll et al., 2017), which performs *in silico* analysis to detect MLST profiles; *rpoB* allelic types (ATs), the belonging to the *panC* gene phylogenetic group; and identification of the closer strain, virulence factors, and AMR genes. Additionally, with the aim to identify the antibiotic resistance genes and plasmids, the draft genome of strains was analyzed using the software ABRicate (Galaxy Version 0.8), which includes different predownloaded databases [ARG-ANNOT (Gupta et al., 2014), NCBI AMRFinderPlus (Feldgarden et al., 2019), CARD (Jia et al., 2017), ResFinder (Zankari et al., 2012), and PlasmidFinder (Carattoli et al., 2014)]. In addition, the species identification was performed also by JSpecieWS online service (Richter et al., 2016)^2^ using pairwise genome comparisons, which measures the average nucleotide identity (ANI) based on BLAST + (ANIb). We selected a total of 50 references genomes: 24 type strains and 26 genomes belonged to *B. cereus* group, of which 18 were defined as effective by Carroll et al. (2017). The draft genomes were also annotated using the software tool Prokka (version 1.13) (Seemann, 2014).

### Nucleotide Sequence Accession Numbers

The draft genomes of *B. cereus* identified have been deposited in GenBank as BioProject PRJNA673333. The numbers of BioSample and accession ID are reported in Table 1.

**TABLE 1:** Characteristics of isolates *Bacillus cereus* s.l.

**ID strain**	**BioSample ID**	**Accession ID**	**Closest strain**	**ST**	**CC**	**rpoB AT**	**MLST *Bacillus cereus***							**panC phylogenetic groups**
							**glp**	**gmk**	**ilv**	**pta**	**pur**	**pyc**	**tpi**	
IZSPB_BC106	SAMN16604380	JADNPV000000000	*B. thuringiensis*_YBT_020	2,163	–	AT0380	19	2	*368*	*333*	*181*	*3*	*275*	III
IZSPB_BC107	SAMN16604381	JADNPW000000000	*B. cereus*_03BB102	163	365	AT0120	44	1	32	16	18	33	24	III
IZSPB_BC108	SAMN16604382	JADNPX000000000	*B. cereus*_Rock1_15	73	142	AT0092	13	8	9	14	9	12	31	IV
IZSPB_BC109	SAMN16604383	JADNPY000000000	*B. cereus_*m1293	1066	205	AT0125	19	2	31	5	19	3	91	III
IZSPB_BC110	SAMN16604384	JADNPZ000000000	*B. cereus_*ATCC_10987	1,032	–	AT0481	117	4	123	118	43	6	3	III
IZSPB_BC111	SAMN16604385	JADNQA000000000	*B. thuringiensis_*T13001	24	–	AT0092	12	8	9	14	11	12	10	IV
IZSPB_BC112	SAMN16604386	JADNQB000000000	*B. thuringiensis_*T03a001	**2,096**	–	AT0154	91	8	14	11	2	36	7	IV
IZSPB_BC114	SAMN16604387	JADNQC000000000	*B. cereus_*m1293	462	–	AT0125	19	2	21	5	19	3	91	III
IZSPB_BC115	SAMN16604388	JADNQD000000000	*B_cereus_*Rock3_42	1,284	–	AT0463	247	1	83	1	239	37	43	III
IZSPB_BC210	SAMN16604389	JADNQE000000000	*B_cereus_*03BB102	365	–	AT0120	34	1	32	1	18	33	24	III
IZSPB_BC211	SAMN16604390	JADNQF000000000	*B_cereus_*ATCC_10987	1,032	–	AT0481	117	4	123	118	43	6	3	III
IZSPB_BC212	SAMN16604391	JADNQG000000000	*B_cereus_*Rock1_15	73	142	AT0092	13	8	9	14	9	12	31	IV
IZSPB_BC213	SAMN16604392	JADNQH000000000	*B_cereus_*03BB102	365	–	AT0120	34	1	32	1	18	33	24	III
IZSPB_BC214	SAMN16604393	JADNQI000000000	*B_cereus_0*3BB102	365	–	AT0120	34	1	32	1	18	33	24	III
IZSPB_BC215	SAMN16604394	JADNQJ000000000	*B_thuringiensis_*T13001	24	–	AT0092	12	8	9	14	11	12	10	IV
IZSPB_BC216	SAMN16604395	JADNQK000000000	*B_thuringiensis_*T13001	24	–	AT0092	12	8	9	14	11	12	10	IV
IZSPB_BC217	SAMN16604396	JADNQL000000000	*B_cereus_*03BB102	365	–	AT0120	34	1	32	1	18	33	24	III

**Sequence type, clonal complex, rpoB allele type, MLST allele, and panC clade of B. cereus s.l. isolates. ST, sequence type; CC, clonal complex; AT, allele type. The sequence type in bold represent a new sequence type. The draft genomes were deposited as BioProject PRJNA673333.**

## Results

Seventeen *B. cereus* s.l. isolated from human blood cultures of 17 patients were studied. MALDI-TOF mass spectrometry (MS) identified all strains as *B. cereus*. The draft genome sequence of the investigated *B. cereus* group isolates consisted of an average of 116 contigs comprising approximately 5,585,000 bp, with almost 5,737 predicted coding region sequences. The average coverage was estimated at ∼68.6*X*. The overall G + C content of 17 isolates was 35%. All data for each isolate were collected in Supplementary Tables 1, 2. The *rpoB* sequence revealed seven distinct ATs (Table 1): AT0380, AT0120, AT0092, AT0125, AT0481, AT0154, AT0125, and AT0463. The sequence of *panC* gene revealed that the isolates belonged to two phylogenetic group: clade III (IZSPB_BC106; IZSPB_BC107; IZSPB_BC109; IZSPB_BC110; IZSPB_BC114; IZSPB_BC115; IZSPB_BC210; IZSPB_BC211; IZSPB_BC213; IZSPB_BC214; IZSPB_BC217) and clade IV (IZSPB_BC108; IZSPB_BC111; IZSPB_BC112; IZSPB_BC212; IZSPB_BC215; IZSPB_BC216) (Table 1). Analysis of MLST genes from our isolate showed different allelic combinations. In particular, 10 different STs were identified (Table 1), one of which (ST2096) resulted in a new ST and was submitted to the online MLST database^3^. Computational analysis performed by BTyper tool confirmed that our 17 isolates belonged to *B. cereus* group. In particular, among them, 12 (IZSPB_BC107, IZSPB_BC108, IZSPB_BC109, IZSPB_BC110, IZSPB_BC114, IZSPB_BC115, IZSPB_BC210, IZSPB_BC212, IZSPB_BC213, IZSPB_BC214, and IZSPB_BC217) were most closely to the type strain of *B. cereus* sensu stricto (*B. cereus* s.s.), and five (IZSPB_BC106, IZSPB_BC111, IZSPB_BC112, IZSPB_BC215, and IZSPB_BC216) were most closely to the type strain of *B. thuringiensis* (Table 1). Additionally, we performed species identification using 50 *B. cereus* species, including 26 type strains, using ANI by the online available service JSpacesWS. The results obtained showed a similar species attribution as provided by BTyper, with some exceptions: only two isolates (IZSPB_BC106 and IZSPB_BC112) were predicted as closer to *B. thuringiensis*, whereas the remaining isolates were all predicted as *B. cereus* s.s. (Supplementary Table 3). The presence of virulence factor genes was assessed by BTyper tool that identified a total of 28 genes (Table 2). Among them, 13 genes were identified in all of the isolates (100%; 17/17): two genes that codified for cereolysin proteins (*cerA* and *cerB*), two enterotoxin genes (*entA* and *entFM*), two immune inhibitor A precursor genes (*inhA1* and *inhA2*), the gene cluster of non-hemolytic enterotoxin (*nheA*, *nheB*, and *nheC*), two genes (*bpsE* and *bpsH*) of the gene cluster of exo-polysaccharide, the sphingomyelinase C gene (*sph*), and the phospholipase C (*plcB*). The pleiotropic regulator (PlcR) of extracellular virulence factor gene (*plcR*) was identified in all of our isolates, although the nucleotidic sequence of this gene matched with different *B. cereus* group species (Table 2). The genes *clo* and *plcA* were present in 94% (16/17) of isolates. The gene *bpsF* was present in 88% (15/17) of isolates; the gene *cytK2* was identified in 59% (10/17) of isolates; the gene *bpsD* was present in 41% (7/17) of isolates. The cluster genes of enterotoxins, hemolysin BL (*hblA*, *hblB*, *hblC*, and *hblD*) and the gene *hlyR*, were identified in 29% (5/17) of isolates (Table 2). Additionally, the annotation of the draft genome was performed for each isolate by the software Prokka, and a total of ∼5,630 genes were annotated (data not shown). Among them, 23 of 32 genes potentially involved in biofilm formation were identified in our isolates; in particular, among these genes, 17 were identified in 100% (17/17), 3 were identified in 97% (16/17), 1 was identified in 18% (3/17) of isolates, and 2 were identified in 12% (2/17) of isolates (Table 3). A total of 12 AMR genes were identified in the genome sequence of our isolates including (Table 4) the following: the vancomycin resistance genes: *Gly-vanR-M*, *Gly-vanZF-Pp*, and *vanR-B*, were identified in 100% (17/17), 88% (15/17), and 12% (2/17) of isolates, respectively; the beta-lactamase resistance genes: *BLA-1* and *BLA-2* and blaZ_12, were identified in 100% (17/17) and 6% (1/17) of isolates, respectively; the fosfomycin resistance gene: fosBx1, was identified in 100% (17/17) of isolates; the macrolide-lincosamide-streptogramin (*MLS-lsaB*) and the virginiamycin acetyltransferase (*vat-E*) were both identified in 41% (7/17) of isolates; the macrolide 2′-phosphotransferase II (*mph-B*) was identified in 35% (6/17) of isolates; the tetracycline resistance gene (*tetL*) and the resistance to macrolides, lincosamides, and streptogramin b (*erm-C*) were identified in 6% (1/17) of isolates, respectively. In addition to the *in silico* analysis, the antimicrobial susceptibility of *B. cereus s. l.* isolates to 15 antibacterial agents was determined, and the results are shown in Table 5. Among beta-lactam antibiotic class, only penicillin G resistance was confirmed in 100% (17/17) of isolates; eight isolates were resistant to ceftriaxone, whereas nine isolates showed intermediate resistance; six isolates were resistant, and 11 showed intermediate resistance to cefotaxime. Resistance to trimethoprim was observed in 100% of the isolates. The isolate IZSPB_BC210 showed resistance to clindamycin, whereas the remaining isolates showed intermediate resistance (24%; 4/17) or resulted susceptible (71%; 12/17). We found intermediate resistance to tetracycline and erythromycin in 24% (4/17) of isolate and to rifampicin in 12% (2/17) of isolates. All the isolates were susceptible to gentamicin, amoxicillin, chloramphenicol, vancomycin, linezolid, ciprofloxacin, and doxycycline.

**TABLE 2:** Virulence factors of clinical isolates of *Bacillus cereus* s.l.

**Virulence gene**	**IZSPB_BC106**	**IZSPB_BC107**	**IZSPB_BC108**	**IZSPB_BC109**	**IZSPB_BC110**	**IZSPB_BC111**	**IZSPB_BC112**	**IZSPB_BC114**	**IZSPB_BC115**
Toxin									
*cerA*	95.05/100	95.76/100	100/100	95.05/100	95.05/100	100/100	100/100	95.05/100	95.05/100
*cerB*	87.45/93.39	92.02/71.47	95.18/93.39	87.78/93.39	87.46/93.39	94.86/93.39	94.86/93.39	87.78/93.39	91.41/87.39
*clo*	95.48/100	96.27/100	99.21/100	95.78/97.84	95.48/100	99.41/100	99.61/100	95.28/100	–/–
*entA*	94.93/100	94.26/100	99.32/100	94.93/100	94.93/100	99.32/100	97.97/100	94.93/100	93.58/100
*entFM*	95.58/100	93.26/100	97.44/100	94.42/100	95.58/100	97.44/100	91.4/100	94.88/100	92.56/100
*nheA*	97.40/100	96.89/100	99.74/100	97.41/100	97.41/100	99.74/100	98.96/100	97.41/100	96.89/100
*nheB*	99.25/100	99.50/100	100/100	99.5/100	99.25/100	100/100	99.5/100	99.5/100	99.25/100
*nheC*	97.497/100	94.43/100	99.72/100	95.54/100	97.49/100	100/100	99.16/100	95.54/100	94.43/100
*cytK2*	–/–	–/–	98.78/97.32	96.73/100	–/–	100/100	99.4/100	–/–	97.62/100
*hblA*	–/–	–/–	99.20/100	–/–	–/–	99.2/100	98.93/100	–/–	–/–
*hblB*	–/–	–/–	99.79/100	–/–	–/–	98.5/100	98.71/100	–/–	–/–
*hblC*	–/–	–/–	98.18/100	–/–	–/–	98.18/100	96.81/100	–/–	–/–
*hblD*	–/–	–/–	99.75/100	–/–	–/–	99.75/100	99.75/100	–/–	–/–
*hlyR*	–/–	–/–	–/–	–/–	–/–	73.13/100	–/–	–/–	99.5/100
Enzyme									
*inhA1*	94.03/100	96.32/100	99.87/100	96.07/100	94.04/100	99.87/100	96.07/100	96.07/100	96.32/100
*inhA2*	96.49/100	96.10/100	99.62/100	97/100	96.5/100	100/100	99.12/100	96.62/100	96.37/100
*sph*	97.92/100	99.22/100	92.01/100	99.11/100	97.93/100	92.31/100	91.42/100	99.11/100	99.7/100
*bpsD*	–/–	–/–	70.21/84.3	–/–	–/–	65.22/92.83	60.99/100	68.09/84.3	–/–
*bpsE*	89.16/93.89	89.89/93.90	90.29/94.24	79.79/95.59	89.17/93.9	88.85/94.24	88.85/94.24	89.57/94.24	87.36/93.90
*bpsF*	50/97.60		51.96/97.61	–/–	50/97.61	51.22/98.09	50.73/98.09	50/97.61	–/–
*bpsH*	67.33/98.68	66.78/100.00	78.95/100	67.32/100.66	67.33/98.68	79.28/100	79.61/100	79.28/100	67.65/100.66
Lipase									
*plcA*	93.31/100	94.83/100	100/100	94.53/100	93.31/100	100/100	97.26/100	94.53/100	94.53/100
*plcB*	95.05/100	95.76/100	100/100	95.05/100	95.05/100	100/100	100/100	95.05/100	95.05/100
Regulation									
*plcR* (*B. cereus* NC7401)	99.64/100	100/100	–/–	99.65/100	99.65/100	–/–	88.34/99.3	99.65/100	–/–
*plcR* (*B. thuringiensis*)	–/–	–/–	–/–	–/–	–/–	–/–	–/–	–/–	100/100
*plcR* (*B. cereus* ATCC 14579)	–/–	–/–	100/100	–/–	–/–	99.65/100	–/–	–/–	–/–
*plcR* (*B. anthracis*)	–/–	–/–	–/–	–/–	–/–	–/–	–/–	–/–	–/–

**Virulence gene**	**IZSPB_BC210**	**IZSPB_BC211**	**IZSPB_BC212**	**IZSPB_BC213**	**IZSPB_BC214**	**IZSPB_BC215**	**IZSPB_BC216**	**IZSPB_BC217**

Toxin								
*cerA*	95.05/100	95.05/100	100/100	95.05/100	95.05/100	100/100	100/100	95.05/100
*cerB*	91.41/87.39	88.42/93.39	95.18/93.39	91.41/87.39	91.41/87.39	94.86/93.39	94.86/93.39	91.41/87.39
*clo*	95.87/100	96.86/100	99.21/100	95.87/100	95.87/100	99.41/100	99.41/100	95.87/100
*entA*	93.58/100	95.27/100	99.32/100	93.58/100	93.58/100	99.32/100	99.32/100	93.58/100
*entFM*	92.56/100	95.35/100	97.44/100	92.56/100	92.56/100	97.44/100	97.44/100	92.56/100
*nheA*	96.11/100	97.15/100	99.74/100	96.11/100	96.11/100	99.74/100	99.74/100	96.11/100
*nheB*	99.5/100	99.5/100	100/100	99.5/100	99.5/100	100/100	100/100	99.5/100
*nheC*	94.71/100	94.71/100	99.72/100	94.71/100	94.71/100	100/100	100/100	94.71/100
*cytK2*	–/–	96.73/100	98.78/97.32	–/–	–/–	100/100	100/100	–/–
*hblA*	–/–	–/–	99.2/100	–/–	–/–	99.2/100	99.2/100	–/–
*hblB*	–/–	–/–	99.79/100	–/–	–/–	98.5/100	98.5/100	–/–
*hblC*	–/–	–/–	98.18/100	–/–	–/–	98.18/100	98.18/100	–/–
*hblD*	–/–	–/–	99.75/100	–/–	–/–	99.75/100	99.75/100	–/–
*hlyR*	–/–	–/–	73.13/100	–/–	–/–	73.13/100	73.13/100	–/–
Enzyme								
*inhA1*	96.19/100	95.56/100	99.87/100	96.19/100	96.19/100	99.87/100	99.87/100	96.19/100
*inhA2*	96.25/100	96.37/100	99.62/100	96.25/100	96.25/100	100/100	100/100	96.25/100
*sph*	99.7/100	97.93/100	92.01/100	99.7/100	99.7/100	92.31/100	92.31/100	99.7/100
*bpsD*	–/–	–/–	70.21/84.3	–/–	–/–	65.22/92.83	65.22/92.83	–/–
*bpsE*	88.45/93.90	88.81/93.9	90.29/94.24	88.45/93.9	88.45/93.90	88.85/94.24	88.85/94.24	88.45/93.9
*bpsF*	50.49/97.61	51.22/98.09	51.96/97.61	50.49/97.61	50.49/97.61	51.22/98.09	51.22/98.09	50.49/97.61
*bpsH*	66.78/100	67.43/100	78.95/100	66.78/100	66.78/100	79.28/100	79.28/100	66.78/100
Lipase								
*plcA*	94.53/100	94.83/100	100/100	94.53/100	94.53/100	100/100	100/100	94.53/100
*plcB*	95.05/100	95.05/100	100/100	95.05/100	95.05/100	100/100	100/100	95.05/100
Regulation								
*plcR* (*B.cereus* NC7401)	–/–	–/–	–/–	–/–	–/–	–/–	–/–	–/–
*plcR* (*B. thuringiensis*)	100/100	–/–	–/–	100/100	100/100	–/–	–/–	100/100
*plcR* (*B. cereus* ATCC 14579)	–/–	–/–	100/100	–/–	–/–	99.65/100	99.65/100	–/–
*plcR* (*B. anthracis*)	–/–	97.89/100	–/–	–/–	–/–	–/–	–/–	–/–

**The table reports the identity and coverage percentages for each virulence gene (Id%/Cov%).**

**TABLE 3:** Genes that can play a role in biofilm formation detected in clinical isolates of *Bacillus cereus* s.l.

**Gene**	**IZSPB_ BC106**	**IZSPB_ BC107**	**IZSPB_ BC108**	**IZSPB_ BC109**	**IZSPB_ BC110**	**IZSPB_ BC111**	**IZSPB_ BC112**	**IZSPB_ BC114**	**IZSPB_ BC115**	**IZSPB_ BC210**	**IZSPB_ BC211**	**IZSPB_ BC212**	**IZSPB_ BC213**	**IZSPB_ BC214**	**IZSPB_ BC215**	**IZSPB_ BC216**	**IZSPB_ BC217**
*xerC*	+	+	+	+	+	+	+	+	+	+	+	+	+	+	+	+	+
*clpY*	+	+	+	+	+	+	+	+	+	+	+	+	+	+	+	+	+
*clpQ*	−	−	−	−	−	−	−	−	−	−	−	−	−	−	−	−	−
*codY*	+	+	+	+	+	+	+	+	+	+	+	+	+	+	+	+	+
*purA*	+	+	+	+	+	+	+	+	+	+	+	+	+	+	+	+	+
*purB*	+	+	+	+	+	+	+	+	+	+	+	+	+	+	+	+	+
*purC*	+	+	+	+	+	+	−	+	+	+	+	+	+	+	+	+	+
*purD*	+	+	+	+	+	+	+	+	+	+	+	+	+	+	+	+	+
*purE*	+	+	+	+	+	+	+	+	+	+	+	+	+	+	+	+	+
*purF*	+	+	+	+	+	+	+	+	+	+	+	+	+	+	+	+	+
*purH*	+	+	+	+	+	+	+	+	+	+	+	+	+	+	+	+	+
*purK*	+	+	+	+	+	+	+	+	−	+	+	+	+	+	+	+	+
*purL*	+	+	+	+	+	+	+	+	+	+	+	+	+	+	+	+	+
*purM*	+	+	+	+	+	+	+	+	+	+	+	+	+	+	+	+	+
*purN*	+	+	+	+	+	+	+	+	+	+	+	+	+	+	+	+	+
*purQ*	+	+	+	+	+	+	+	+	+	+	+	+	+	+	+	+	+
*purS*	+	+	−	+	+	+	+	+	+	+	+	+	+	+	+	+	+
*yezC*	−	−	−	−	−	−	−	−	−	−	−	−	−	−	−	−	−
*epsA*	−	−	−	−	−	−	−	−	−	−	−	−	−	−	−	−	−
*epsB*	−	−	−	−	−	−	−	−	−	−	−	−	−	−	−	−	−
*epsD*	+	−	−	+	−	−	−	−	−	−	+	−	−	−	−	−	−
*epsG*	+	−	−	+	−	−	−	−	−	−	−	−	−	−	−	−	−
*epsK*	−	−	−	−	−	−	−	−	−	−	−	−	−	−	−	−	−
*epsM*	+	−	−	+	−	−	−	−	−	−	−	−	−	−	−	−	−
*epsO*	−	−	−	−	−	−	−	−	−	−	−	−	−	−	−	−	−
*sipW*	+	+	+	+	+	+	+	+	+	+	+	+	+	+	+	+	+
*tasA*	+	+	+	+	+	+	+	+	+	+	+	+	+	+	+	+	+
*sinR*	+	+	+	+	+	+	+	+	+	+	+	+	+	+	+	+	+
*sinI*	−	−	−	−	−	−	−	−	−	−	−	−	−	−	−	−	−
*tapA*	−	−	−	−	−	−	−	−	−	−	−	−	−	−	−	−	−
*aad*	−	−	−	−	−	−	−	−	−	−	−	−	−	−	−	−	−
*sigB*	+	+	+	+	+	+	+	+	+	+	+	+	+	+	+	+	+

**TABLE 4:** Presence of antimicrobial resistance genes.

**AMR gene**	**IZSPB_ BC106**	**IZSPB_ BC107**	**IZSPB_ BC108**	**IZSPB_ BC109**	**IZSPB_ BC110**	**IZSPB_ BC111**	**IZSPB_ BC112**	**IZSPB_ BC114**	**IZSPB_ BC115**	**IZSPB_ BC210**	**IZSPB_ BC211**	**IZSPB_ BC212**	**IZSPB_ BC213**	**IZSPB_ BC214**	**IZSPB_ BC215**	**IZSPB_ BC216**	**IZSPB_ BC217**
(Gly)vanZF-Pp| AF155139| 4339-4959| 621	+	+	+	−	+	+	+	+	+	+	+	+	+	+	+	+	+
(Gly)vanR-M| FJ349556| 982-1680| 699	+	+	+	+	+	+	+	+	+	+	+	+	+	+	+	+	+
vanR-B	+	−	−	−	−	−	−	−	−	−	+	−	−	−	−	−	−
(Bla)bla2| NG_047224| 101-874| 774	+	+	+	+	+	+	+	+	+	+	+	+	+	+	+	+	+
(Bla)BLA-1| AY453161| 501-1430| 930	+	+	+	+	+	+	+	+	+	+	+	+	+	+	+	+	+
blaZ_12	−	−	−	−	−	−	+	−	−	−	−	−	−	−	−	−	−
(Fcyn)fosBx1| NG_050591| 101-517| 417	+	+	+	+	+	+	+	+	+	+	+	+	+	+	+	+	+
Mph (B)	−	+	−	−	−	−	−	−	+	+	−	−	+	+	−	−	+
(MLS)lsa (B)| AJ579365| 4150-5628| 1479	−	−	+	−	−	+	+	−	+	−	−	+	−	−	+	+	−
(Tet)tetL| FN435329| 1-1377| 1377	−	−	−	+	−	−	−	−	−	−	−	−	−	−	−	−	−
vat(E)	−	−	+	−	+	+	−	−	−	−	+	+	−	−	+	+	−
erm(C)	−	−	−	−	−	−	−	−	−	+	−	−	−	−	−	−	−

**TABLE 5:** Antimicrobial resistance pattern of clinical isolates of *Bacillus cereus* s.l.

		**Interpretive Criteria (μg/mL)**			**IZSPB_ BC106**	**IZSPB_ BC107**	**IZSPB_ BC108**	**IZSPB_ BC109**	**IZSPB_ BC110**	**IZSPB_ BC111**	**IZSPB_ BC112**	**IZSPB_ BC114**	**IZSPB_ BC115**	**IZSPB_ BC210**	**IZSPB_ BC211**	**IZSPB_ BC212**	**IZSPB_ BC213**	**IZSPB_ BC214**	**IZSPB_ BC215**	**IZSPB_ BC216**	**IZSPB_ BC217**
**Antibioticrobial agent**	**Range**	**S**	**I**	**R**																	

Gentamicin	0.008– 16	≤ 4	8	≥ 16	S (1)	S (0.5)	S (1)	S (2)	S (1)	S (1)	S (1)	S (1)	S (0.5)	S (0.5)	S (2)	S (0.5)	S (1)	S (1)	S (1)	S (1)	S (1)
Penicillin G	0.06– 128	≤ 0.12	–	≥ 0.25	R (> 128)	R (8)	R (> 128)	R (32)	R (64)	R (> 128)	R (> 128)	R (> 128)	R (16)	R (> 128)	R (64)	R (> 128)	R (> 128)	R (> 128)	R (128)	R (> 128)	R (> 128)
Amoxicillin	0.008– 16	≤ 0.12	–	≥ 0.25	S (0.125)	S (0.125)	S (0.125)	S (0.125)	S (0.125)	S (0.125)	S (0.125)	S (0.06)	S (0.06)	S (0.06)	S (0.125)	S (0.06)	S (0.125)	S (0.06)	S (0.06)	S (0.125)	S (0.06)
Clindamycin	0.008– 16	≤ 0.5	1– 2	≥ 4	S (0.125)	S (0.125)	S (0.25)	S (0.25)	S (0.25)	S (0.50)	S (0.25)	S (0.25)	I (1)	R (> 16)	S (0.50)	I (1)	S (0.50)	S (0.50)	I (1)	I (1)	S (0.50)
Chlo.ramphenicol	0.06– 128	≤ 8	16	≥ 32	S (4)	S (4)	S (2)	S (4)	S (4)	S (4)	S (4)	S (4)	S (4)	S (4)	S (4)	S (2)	S (4)	S (2)	S (2)	S (1)	S (2)
Vancomycin	0.03– 64	≤ 4	–	–	S (2)	S (2)	S (2)	S (2)	S (2)	S (2)	S (2)	S (1)	S (2)	S (2)	S (1)	S (1)	S (2)	S (2)	S (1)	S (1)	S (2)
Linezolid	0.03– 64	≤ 4	–	≥ 8	S (2)	S (1)	S (1)	S (2)	S (2)	S (2)	S (1)	S (2)	S (2)	S (0.5)	S (1)	S (0.5)	S (1)	S (1)	S (1)	S (1)	S (1)
Erythromycin	0.008– 16	≤ 0.5	1– 4	≥ 8	S (0.125)	I (4)	I (4)	S (0.06)	S (0.06)	S (0.06)	S (0.06)	S (0.125)	S (0.5)	I (4)	S (0.06)	I (4)	S (0.5)	S (0.5)	S (0.06)	S (0.25)	S (0.5)
Tetracycline	0.008– 16	≤ 4	8	≥ 16	I (8)	S (4)	I (8)	I (8)	S (4)	S (4)	S (4)	S (1)	S (0.5)	S (0.5)	S (2)	I (8)	S (0.5)	S (0.5)	S (2)	S (2)	S (0.5)
Ciprofloxacin	0.004– 8	≤ 1	2	≥ 4	S (0.06)	S (0.06)	S (0.06)	S (0.06)	S (0.06)	S (0.06)	S (0.06)	S (0.03)	S (0.06)	S (0.06)	S (0.125)	S (0.25)	S (0.25)	S (0.06)	S (0.25)	S (0.125)	S (0.06)
Doxycycline	0.002– 4	≤ 4	8	≥ 16	S (0.06)	S (0.06)	S (0.25)	S (0.25)	S (0.125)	S (0.25)	S (0.25)	S (0.06)	S (0.03)	S (0.03)	S (0.25)	S (0.125)	S (0.06)	S (0.06)	S (0.125)	S (0.125)	S (0.03)
Rifampicin	0.004– 8	≤ 1	2	≥ 4	I (2)	S (0.5)	S (0.5)	I (2)	S (1)	S (1)	S (0.5)	S (1)	S (0.25)	S (0.06)	S (0.125)	S (0.125)	S (0.06)	S (0.125)	S (0.25)	S (0.25)	S (0.25)
Ceftriaxone	0.25– 512	≤ 8	16– 32	≥ 64	I (32)	R (64)	I (32)	I (32)	R (64)	I (32)	R (512)	I (32)	I (32)	R (> 512)	I (32)	I (32)	R (> 512)	R (> 512)	I (32)	R (> 512)	R (> 512)
Cefotaxime	0.25– 512	≤ 8	16– 32	≥ 64	I (32)	I (32)	I (16)	I (32)	I (32)	I (16)	R (128)	I (16)	I (16)	R (128)	I (32)	I (32)	R (128)	R (128)	I (32)	R (128)	R (128)
Trimethoprim	0.06– 128	–	–	≥ 16	R (> 128)	R (> 128)	R (> 128)	R (> 128)	R (> 128)	R (> 128)	R (> 128)	R (> 128)	R (> 128)	R (> 128)	R (> 128)	R (> 128)	R (> 128)	R (> 128)	R (> 128)	R (> 128)	R (> 128)

**All parameters were interpreted according to the NCCLS antimicrobial susceptibility standards for staphylococci. S, susceptible; I, intermediate; R, resistant. The breakpoints (μg/mL) for Staphylococcus spp. were used for linezolid, doxycicline and trimethoprim according to CLSI guidelines M100-S24 (2014), whereas for the other antimicrobials, the interpretative criteria for Bacillus spp. were used according to CLSI guidelines M45-A2 (2011). The MIC value (μg/mL) is shown in parentheses. S, susceptible; I, intermediate; R, resistant.**

Two plasmid replicons (*rep*) were detected: *rep12* belonging to a cryptic plasmid pBMB67 in IZSPB_BC107, IZSPB_BC108, and IZSPB_BC212, and *rep22* belonging to pUB110 in IZSPB_BC109 and IZSPB_BC210; additionally, in IZSPB_BC210, it was found repUS12 belonging to pUB110, also. Interestingly, the large plasmid that carries *cry* genes was not identified in none of the isolates that were predicted as closely to *B. thuringiensis*.

## Discussion

*Bacillus* species are widely distributed in nature and can colonize hospital environments; indeed, there is evidence that strains of *B. cereus* were found on the hands of nursing staff, in balloons used for manual ventilation and near ventilation system outlets (Kuroki et al., 2009). Recent reports suggest that *B. cereus* s.l. can cause nosocomial bacteremia via catheter-related infections caused by the formation of biofilm on biomedical devices (Jensen et al., 2003; Dohmae et al., 2008). Genetically, *B. cereus* s.s. is closely related to *B. thuringiensis* that is used extensively worldwide as pesticide in forestry and agriculture (Zhu et al., 2015). The aim of this study was to better understand the genetic characteristics of clinical *B. cereus* group isolates. In the past, several surveys using a variety of methods for detection of *B. cereus* group have been performed. However, some of them failed to discriminate between *B. cereus* group members at the species level (Seemann, 2014; Caamaño-Antelo et al., 2015; Zhu et al., 2015; Richter et al., 2016; Raymond and Federici, 2017). Generally, the identification and typing of *B. cereus* s.l. are based on MLST^4^ or on the identification of virulence genes using PCR. Nevertheless, these methods are too expensive, time consuming, and labor intensive, and sometimes, because of the high genomic similarity within the group, they failed to identify or type correctly *B. cereus* spp. To date, different innovative techniques are available. Among these, MALDI-TOF MS is becoming an increasingly useful method for the rapid identification of bacteria and fungi. In fact, compared to conventional methods (phenotypic, genotypic, and immunological tests), this technology is fast and cheap and, for clinically significant bacteria, can provide accurate and reliable results from a single isolated colony within minutes. In our study, we compared the performance of two approaches for the capability to assign the isolates to the correct species: the MALDI-TOF MS and the ANIBlast method using the whole genome; for the last approach, we used two tools, BTyper and JSpeciesWS. Although both methods identified our isolates as *B. cereus* group members, the ANIBlast method was performing better, as it was able to identify two ANI groups, which included *B. cereus* s.s. and *B. thuringiensis* species, respectively. Even though the species predicted was the same using both ANIBlast tools, a different species attribution among our isolates was found: when we used Btyper, we found five *B. thuringiensis*, whereas when we used online available ANI Blast calculator, we found two B. *thuringiensis*; in both predictions, the remaining isolates were predicted as close to *B. cereus* s.s. However, *B. thuringiensis* virulence–associated genes and plasmids were not detected in any of our isolates. However, *B. thuringiensis* clones lacking Cry toxin have been described elsewhere (Zhu et al., 2015; Fayad et al., 2019) and defined as *B. thuringiensis-like* (Fayad et al., 2019). Thus, our results showed the limit in using the MALDI-TOF MS–based identification method, perhaps because some *B. cereus* group members, especially *B. cereus* s.s. and *B. thuringiensis*, do not have sufficient differences in their protein sequences, as they are genetically very similar (Guinebretière et al., 2008; Zheng et al., 2017).

With the aim of classifying potential pathogenic microorganisms quickly and effectively, WGS of 17 *B. cereus* group clinical isolates was performed. Based on the *rpoB* sequence, we identified seven different ATs, which showed a very high similarity (100%) to *B. cereus* s.s. Analysis sequence of *panC* gene revealed that the isolates belonged to phylogenetic groups III and IV. Interestingly, both groups include *B. cereus* s.s. isolates from hospitals and from patients (EFSA Panel on Biological Hazards, 2009), as well as more foodborne poisoning strains as reported in Guinebretière et al. (2008). The MLST analysis showed that the 17 isolates belonged to seven different STs, suggesting that the ability of *B. cereus* strains to cause human infection is not restricted to a specific ST or clonal group. About these, two sequence types, ST163 and ST73, were previously described as etiological cause of respiratory infection and pneumonia cases in Japan, respectively (Beecher and Wong, 2000). Because of the rarity of *B. cereus* infection and a paucity of genetic information, it is unclear whether particular genetic elements are associated with specific clinical manifestations. Generally, the pathogenicity of *B. cereus* has been associated with toxin production and putative virulence factors, such as enzymes and proteases, which are still poorly explored (EFSA Panel on Biological Hazards, 2016). Among the virulence factors, the toxins, such as hemolytic enterotoxin HBL, non-hemolytic enterotoxin NHE, cytotoxin K, and enterotoxin FM, have been associated with diarrheal diseases (Granum et al., 1999; Beecher and Wong, 2000; Lund et al., 2000; Hansen and Hendriksen, 2001; Fagerlund et al., 2004; Sergeev et al., 2006). The metalloprotease (*inhA*), the exo-polysaccharide (bpsX-H), and the phospholipases *sph* genes are important virulence factors as they make bacilli enable to escape innate and adaptive immune responses during infective phases (González-Zorn et al., 1999; Ramarao and Lereclus, 2005; Guillemet et al., 2010; Oh et al., 2011; Oda et al., 2012). Moreover, *sph* exhibits potent hemolytic activity; thus, it has been associated as a virulence factor to septicemic infections (Oda et al., 2013). Also, the *CytK-2* protein is a hemolytic toxin, and it is able to form pores in planar lipid bilayers and to cause toxic effects on human intestinal cells (Fagerlund et al., 2004). Other enterotoxigenic factors are enterotoxin FM (*entFM*) and enterotoxin A (*entA*) genes. There is evidence that suggest *entFM* contributes to the severity of diarrheal illness (Castiaux et al., 2016). The phosphatidylcholine-preferring phospholipase and the sphingomyelinase constitute the hemolytic cereolysin AB complex; both play an important cytotoxic role in many infections, considering that they show the ability to hydrolyse membrane phospholipids (Titball, 1993). The physiological roles of the bacterial enzymes are not well understood, although it has been suggested that the phosphatidylinositol-specific phospholipase C (PI-PLC) are virulence factors in the human pathogens *Listeria monocytogenes* and *S. aureus* (Gässler et al., 1997). We also identified PlcR, a regulation protein, which is a well-known pleiotropic regulator of genes related to pathogenicity (Salamitou et al., 2000). Although the virulence factors associated with clinical non-gastrointestinal diseases are unclear, in our clinically isolates, we found the contemporary presence of genes encoding to *NHE*, *entA*, *entFM*, *sph*, *cerA*, *cerB*, *inhA*, *plcA*, and *plcB* virulence factors, in co-presence of the PlcR (Salamitou et al., 2000). Our findings show the high toxigenic potential of these bacteria. Interestingly, the isolates that in addition carry the genes that encoded enterotoxin hemolysin BL might be more virulent (Salamitou et al., 2000), whereas the absence of one of the virulence factors here described is not necessarily associated with a low pathogenicity power of *Bacillus* isolates. This assertion might be due to several reasons: in literature, a cytotoxic strain, *B. cereus* ATCC 10987, was reported that lacked the *HBL* operon, but produced a large amount of the *NHE* mRNA and exhibited a strong cytopathogenic activity in Vero cells (Lindbäck et al., 1999); the absence of one cytotoxic component may be compensated by the expression of other PlcR-regulated factors, which are still unknown. The gene *PlcR* plays an important role also in the biofilm formation (Ryu and Beuchat, 2005; Hsueh et al., 2006). The biofilm consists of a complex community and makes the *B. cereus* members group capable to colonize different environments (Majed et al., 2016). The isolates that possess *Plc*R may take advantage both in virulence genes regulation and in biofilm formation. Previous studies suggested that certain *B. cereus* strains were able to form different types of biofilms, either submerged, bottom-surface attached biofilms, floating pellicles, or pellicles attached to the side surfaces of the glass tubes. Different types of biofilms may require activities from different genetic determinants (Wijman et al., 2007; Caro-Astorga et al., 2015; Gao et al., 2015; Yan et al., 2017). In the closely related species *Bacillus subtilis*, an operon has been described, including three genes (*tasA*, *tapA*, *sipW*), which is required to form the biofilm (Candela et al., 2018). The transcription of these genes is promoted by *Sin*I and repressed by *SinR* (Kearns et al., 2005). Two orthologs of *tasA* have been described: the first is also named *tasA*, and it is found downstream of the signal peptidase gene *sipW*, in the *SinR*-regulated bicistronic operon *sipW*-*tasA* (Pflughoeft et al., 2011; Fagerlund et al., 2014; Caro-Astorga et al., 2015); the second is named *calY* and is located downstream from *sipW*-*tasA* (Caro-Astorga et al., 2015). In *B. cereus*, both *CalY* and *Tas* polymerize to form fibers in the matrix biofilm (Caro-Astorga et al., 2015). *TapA* contributes to the beginning and growth of the *tasA* fibers (Romero et al., 2014), but the strains that lack this gene contain all elements required for fiber assembly (Caro-Astorga et al., 2015). All of our isolates carried *calY*, *tasA*, and *sipW* genes. Although our isolate did not carry the gene *tapA*, which encodes for the accessory protein, the presence of the three genes mentioned above may be sufficient for the biofilm production. This statement is in accordance with a previous study where a similar condition was found (Caro-Astorga et al., 2015).

Another gene associated with biofilm formation in *B. cereus* is represented by global regulator *CodY* (Lindbäck et al., 2012). *CodY* gene resides in an operon with other genes, such as *xerC*, *clpY*, and *clpQ* (Slack et al., 1995). Altogether, these genes play a role in pellicle biofilm formation and swarming motility (Yan et al., 2017). Additionally, in *B. subtilis*, there are several *esp* genes (*epsA-O*) that are strongly expressed during biofilm formation (Vlamakis et al., 2013). We did not find *eps* gene cluster in our isolates, with few exceptions; a previous study noticed that in *B. cereus*, these genes did not appear to be important for pellicle formation, despite the important role described in equivalent product for *B. subtilis* (Gao et al., 2015). The *pur* gene cluster, including 11 genes, was required for purine biosynthesis (Vilain et al., 2009; Yan et al., 2017). Biofilm of several bacterial species, including *B. cereus*, has previously been shown to contain extracellular DNA as an integral component of extracellular polymeric substance (Vilain et al., 2009). In some bacteria, including *B. subtilis*, the alternative sigma factor σ^*B*^, which is encoded by *sigB* gene, plays a role in stress conditions. *SigB* gives to the bacteria the ability to resist multiple stresses (van Schaik et al., 2004; Hecker et al., 2007). In the studied isolates, we investigated the presence of the determinants that may be related to biofilm formation, although we did not assess this ability *in vitro*. This aspect has a clinical importance, considering that the biofilm formed by pathogenic species is often associated with hospital-acquired infection (Lindbäck et al., 2012). Intriguingly, the majority of the virulence factors here identified appeared to be evenly distributed among *B. cereus* s.s. isolates, as well as strain close to type *B. thuringiensis* strains. This suggests that investigating the set of virulence factors regardless of right species identification could be more important to define the pathogenic power of strains belonging to *B. cereus* group.

Considering the emergence of antibiotic-resistant *B. cereus* strains, which may result in the failure of antibiotic treatment, it became highly relevant for public health to know the attitude of antibiotic resistance in *B. cereus*. In this study, we performed either *in silico* and *in vitro* analyses. Typically, *B. cereus* is resistant to penicillin G or other beta-lactam antibiotics (Citron and Appleman, 2006), and we found that all the isolates were resistant (penicillin G) and resistant or moderately resistant (i.e., ceftriaxone and cefotaxime) to beta-lactam antibiotics and carried genes related to this resistance. Similar to the results of other studies (Luna et al., 2007; Park et al., 2009; Raymond et al., 2010), our isolates showed susceptibility to ciprofloxacin, chloramphenicol, gentamicin, linezolid, and doxycycline. In some isolates, we identified two genes (*ermC* and *mphB*) that confer resistance to macrolide drug family, but when we compared this result with phenotypic test, only for one isolate that the results agreed. All isolates carried genes associated with vancomycin resistance, but all isolates resulted susceptible to the phenotypic test. Additionally, all the isolates harbored the Fcyn-fosBx1 gene that probably confers resistance to fosfomycin, but we do not have phenotypic information, as there are no references on the interpretation. Similarly, we identified in two isolates the puB110 plasmid, which has been associated with kanamycin resistance, but no phenotypic information was available. On the contrary, the phenotypic test revealed that all isolates were resistant to trimethoprim, but in the genome of each isolate, we did not detect a specific genetic determinant that could clarify the resistance. This can be explained in many ways: there are several genetic mechanisms for resistance to a single antimicrobial agent; there are genetic mechanisms that can give rise to antibiotic resistance, i.e., mutations or acquisition of new genetic material (plasmids). There is insufficient knowledge about all genetic variations leading to reduced susceptibility for a given antimicrobial agent (Ellington et al., 2017). Thus, the AMR genomic data related are never static, as the genetic information is always moving. In conclusion, our AMR results underline the importance of combining the information detected by genome to those detected by phenotypic test and the necessity to update continually the databases used to this aim.

Taken together, this study suggests that the identification of *B. cereus* and of the several toxins produced by this bacterium should be considered essential to assess the risk for human health. However, the comprehensive risk characterization in clinical infections is difficult because of the underestimation of the risk, as the clinical laboratories do not necessarily complete species identification considering *B. cereus* as food-borne and/or environmental contaminants. Thus, we analyzed the molecular and genetic data in order to alert clinicians regarding the emerging threat that *B. cereus* can represent in clinical settings. Our results suggest that the analysis of WGS data, followed by appropriate data analysis strategies, could be a highly effective way to evaluate the pathogenic potential of *B. cereus*. The comprehensive molecular characterization of the isolates allowed identifying genetic diversity, as we identified different STs. Interestingly, we identified two members of *B. cereus* group, and the analyses reveal that strain close to *B. thuringiensis*, which lacks of cry plasmid, can carry similar virulence determinants as *B. cereus* s.s. Without specific information about the patients, for clinical treatment and for the human safety, it is essential to ensure an adequate antibiotic therapy. To this aim, our results showed that aminoglycosides, oxazolidinone, and lincosamide might be a good choice for treating *B. cereus* infections; on the opposite, penicillin and third-generation cephalosporins are not recommended. Certainly, more studies on clinical isolates are necessary to collect more information about pathogenicity of these strains with the aim to improve the genomic correlations that will help to identify the most pathogenic strains and take prompt action.

## Data Availability Statement

The datasets GENERATED for this study can be found in NCBI BioProject PRJNA673333.

## Ethics Statement

Ethical approval was not provided for this study on human participants because the samples were collected during the last 3 years. After diagnostic routine, strains resulted from the biological material were stored to be processed for further analysis. No personal data or any other information than the type of material and the result of routine microbiology analysis were collected from each specimen, inhibiting any correlations of these fully anonymized samples to the respective patients. Thus, according to national regulations and the institutional rules for Good Scientific Practice, the requirement for submission to an ethical committee and for obtaining patients’ informed consent was waived. Written informed consent for participation was not required for this study in accordance with the national legislation and the institutional requirements.

## Author Contributions

AP and MM provided substantial contributions to the conception and design of the work and the acquisition, analysis, and interpretation of data and drafting the work. AB, LC, and LD performed the acquisition, analysis, and interpretation of data and drafting the work. VM, GP, and DL performed the acquisition, analysis, and interpretation of data and critical revising of the work for important intellectual content. All authors have approved the final version to be published. All authors are to be accountable for all aspects of the work in ensuring that questions related to the accuracy or integrity of any part of the work are appropriately investigated and resolved.

## Conflict of Interest

The authors declare that the research was conducted in the absence of any commercial or financial relationships that could be construed as a potential conflict of interest.

## Footnotes

1 http://mlstoslo.uio.no/

2 http://jspecies.ribohost.com/jspeciesws/

3 https://pubmlst.org/bcereus/

4 https://pubmlst.org/
